# A matter of timing: harm reduction in learned helplessness

**DOI:** 10.1186/1744-9081-10-41

**Published:** 2014-11-03

**Authors:** Sophie Helene Richter, Alexander Sartorius, Peter Gass, Barbara Vollmayr

**Affiliations:** Animal Models in Psychiatry, Department of Psychiatry and Psychotherapy, Centra, Institute of Mental Health, Medical Faculty Mannheim/Heidelberg University, 68159 Mannheim, Germany; Bernstein Center for Computational Neuroscience, Heidelberg/Mannheim, Germany; Department of Behavioural Biology, Institute of Neuro and Behavioural Biology, University of Münster, Münster, Germany; Translational Imaging, Department of Psychiatry and Psychotherapy, Central Institute of Mental Health, Medical Faculty Mannheim/Heidelberg University, Mannheim, Germany

**Keywords:** Learned helplessness, Reliability, Refinement, Harm reduction, Latency measures, ROC-curves

## Abstract

**Background:**

Learned helplessness has excellent validity as an animal model for depression, but problems in reproducibility limit its use and the high degree of stress involved in the paradigm raises ethical concerns. We therefore aimed to identify which and how many trials of the learned helplessness paradigm are necessary to distinguish between helpless and non-helpless rats.

**Findings:**

A trial-by-trial reanalysis of tests from 163 rats with congenital learned helplessness or congenital non-learned helplessness and comparison of 82 rats exposed to inescapable shock with 38 shock-controls revealed that neither the first test trials, when rats showed unspecific hyperlocomotion, nor trials of the last third of the test, when almost all animals responded quickly to the stressor, contributed to sensitivity and specificity of the test. Considering only trials 3–10 improved the classification of helpless and non-helpless rats.

**Conclusions:**

The refined analysis allows abbreviation of the test for learned helplessness from 15 trials to 10 trials thereby reducing pain and stress of the experimental animals without losing statistical power.

## Findings

### The model of learned helplessness

Originally described by Overmier and Seligman in the sixties [1], the learned helplessness (LH) paradigm has become the most widely studied animal model of depression. It is based on the idea that organisms exposed to inescapable shock in one situation fail to learn to escape shock in a different situation [2]. Meanwhile, stressor (un)controllability effects have been demonstrated in a broad range of species, including rats and mice, and have been shown to extend to a wide range of behavioral and neurochemical consequences [3]. Despite the generality of the LH phenomenon, and its excellent validity two factors may limit its overall acceptance and usefulness: first, difficulties in reliability of the paradigm have repeatedly been discussed [4, 5], and, second, a central feature of the LH procedure, i.e. the stressor uncontrollability, is likely to cause pain and suffering to the animals and as such has moved into the focus of animal welfare regulations. Because of the exceptional translational validity of the model - it is the basis for developing new treatment strategies of treatment-resistant depression [6–8] - an absolute replacement is not possible. We therefore seek for a procedural refinement that goes along with a significant reduction of harm and stress experienced by animals undergoing the procedure [9].

### How to assess learned helplessness in non-human animals

Helpless behavior in the LH paradigm can be assessed and categorized on the basis of either a continuous variable (i.e., the latency to press the lever), or a discrete one (i.e., the number of failures to escape) [1, 2, 10]. Specifically, for the categorization of animals as helpless or not, the so-called failure pattern (FP), i.e. the number of failures to terminate shock, has been demonstrated to be a more reproducible and more reliable measure than the deficit pattern (DP), i.e. the number of failures to terminate shock within the first 20 s of a trial [5, 11]. However, while the use of a discrete variable allows for a clear categorization and distinction between two groups of animals, a continuous variable more fully describes the behavioral performance in the paradigm and allows for also investigating behavioral changes over time. The basic LH paradigm relies on rules of operant conditioning and is based on a series of test trials. Usually, the individual’s performance in these trials is summed up to get a final LH value (i.e. DP, FP, or sum of latencies [1, 2, 5]), thereby overlooking the fact that the behavioral responses may change over time as a result of learning. Thus, the widely favoured categorization approach inevitably goes along with a loss of information that can be avoided using a continuous variable, such as the latency until pressing the lever, and including a trial-by-trial analysis. The aim of the current study was to reassess the procedure for inescapable shock and LH testing, trying to minimize pain, stress and discomfort experienced by the animals during testing, while at the same time working out a proper test protocol that nonetheless allows for reliably discriminating between helpless and non-helpless animals.

All procedures complied with the regulations covering animal experimentation within the EU (European Communities Council Directive 86/609/EEC). They were conducted in accordance with the institutions' animal care and use guidelines and approved by the national and local authorities (Regierungspräsidium Karlsruhe). Moreover, all efforts were made to minimize the number of animals used and the severity of procedures applied in this study.

### Re-thinking the analysis of learned helplessness

In a first step, we reanalysed LH-tests from 163 female and male congenitally helpless (cLH) and non-helpless (cNLH, resistant to the effects of inescapable stress) rats. Originally, these rats were bred from Sprague Dawley rats through selective mating of animals that differed in their susceptibility to develop LH [12, 13]. At the age of nine (male) or ten weeks (female), rats of the cLH and cNLH strains undergo an escape paradigm in operant chambers to confirm the helpless or non-helpless phenotype. Briefly, this rat test version consists of 15 trials, in which an electric foot shock (0.8 mA, 60 s) can be terminated by the animals pressing a bar.

**Figure 1:**
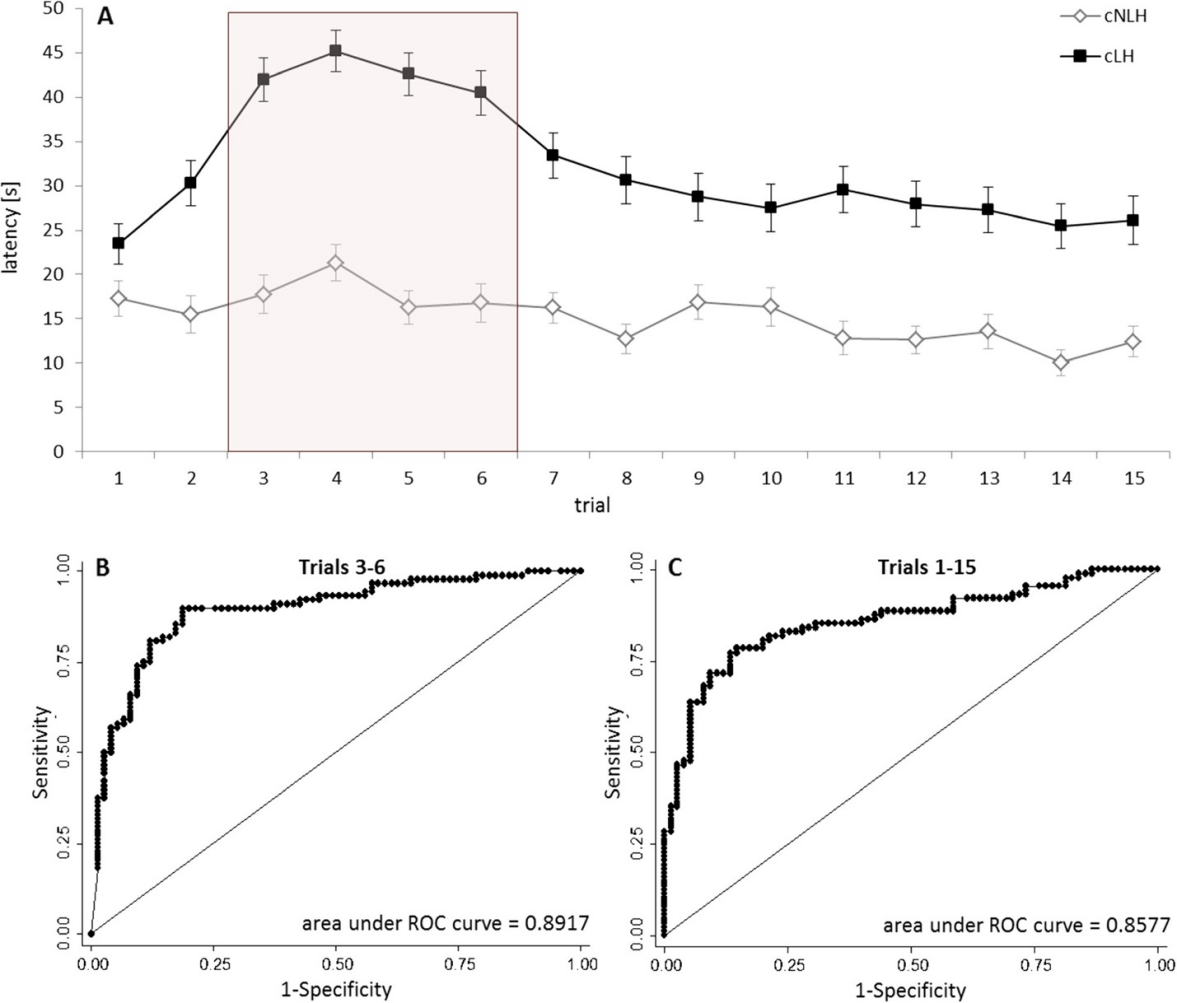
**Reanalysis of data from 163 congenitally helpless (cLH, n = 88) and non-helpless rats (cNLH, n = 75). A**: All rats have been tested for learned helplessness at the age of nine or ten weeks to confirm the helpless or non-helpless phenotype in the escape paradigm. The test consisted of 15 trials in which an electric foot shock (0.8 mA, 60 s) could be terminated by the animals pressing a bar. Latency until pressing the lever is given separately for the strains as means ± SEM. As indicated by the red box, the latency difference between cLH and cNLH was most pronounced between trials 3 to 6. **B** and **C**: Receiver Operating Characteristic (ROC)-curves: In a ROC-curve the true positive rate (sensitivity) is plotted in function of the false positive rate (100-specificity). A test with perfect discrimination (no overlap between the two distributions) has a ROC-curve that passes through the upper left corner (100% sensitivity, 100% specificity). Therefore the closer the ROC curve is to the upper left corner, the higher the overall accuracy of the test [14]. Latency measurements of trials 3 to 6 classified cLH and cNLH rats with at least the same sensitivity and specificity as being helpless or non-helpless as trials 1 to 15 did.

For the present analysis, all LH data from the subsequent generations 72, 73, and 74 of the colonies bred at the Central Institute of Mental Health in Mannheim were pooled and re-analysed with respect to latency measurements (data of males of the generations 72 and 73 have previously been published [14]). Already the descriptive presentation of the latency measures over the time course of the LH-test showed that reactions to shock exposure might be confounded or overlaid by other behavioral processes occurring at the same time (Figure 1A): Upon first exposure to shock all animals reacted with some kind of hyperlocomotive agitation in the box, paralleled by a more or less incidental pressing of the lever. In the subsequent trials, cNLH were characterized by constantly low latencies, showing that they have quickly learned the contingency between bar-pressing and shock termination, while cLH rats showed typical freezing behavior accompanied by higher latencies to terminate the shock. Around trials 6 to 7, latencies started to decrease also in cLH rats, documenting the learning process in these rats. Consequently, the difference between cLH and cNLH rats in the mean latencies to press the lever was most pronounced in the first half of testing, specifically between trials 3 to 6, and then became less apparent in the second half of the trials (Figure 1A). However, to be able to reliably distinguish between helpless and non-helpless animals, it is important to disentangle the specific LH behavior from other behavioral processes, such as hyperlocomotion superimposed during the first trials, or learning, becoming apparent as LH-specific behavior ceases. Therefore, to find out, which trials maximise the discriminative power, we summed up latencies across one to six subsequent trials (bin sizes 1–6), and calculated the area under the ROC-curves (AUC, measure of how well a parameter can distinguish between two groups [15]) for each sliding window (Table 1). As already assumed on the basis of the descriptive analysis (Figure 1A), we found the highest AUC value for trials 3 to 6 (bin size 4, Table 1). Here, the absolute AUC value was even higher (Figure 1B) than the AUC based on all trials (Figure 1C), indicating at least equal sensitivity and specificity using this specific trial window.

**Table 1:** **Area under the Receiver Operating Characteristic (ROC) curves for bin sizes 1 to 6 used to re-analyse data from 163 congenitally helpless (cLH, n = 88) and non-helpless rats (cNLH, n = 75)**

Trials	Bin size 1	Bin size 2	Bin size 3	Bin size 4	Bin size 5	Bin size 6
	AUC ± SEM	AUC ± SEM	AUC ± SEM	AUC ± SEM	AUC ± SEM	AUC ± SEM
1	0,604 ± 0,045	0,648 ± 0,043	0,750 ± 0,038	0,802 ± 0,034	0,856 ± 0,030	0,878 ± 0,027
2	0,678 ± 0,042	0,777 ± 0,037	0,823 ± 0,033	0,870 ± 0,029	0,891 ± 0,026	0,888 ± 0,026
3	0,763 ± 0,038	0,831 ± 0,033	0,880 ± 0,029	0,892 ± 0,026	0,888 ± 0,026	0,884 ± 0,027
4	0,782 ± 0,037	0,848 ± 0,031	0,866 ± 0,029	0,861 ± 0,030	0,856 ± 0,031	0,846 ± 0,032
5	0,809 ± 0,034	0,845 ± 0,031	0,842 ± 0,032	0,841 ± 0,032	0,829 ± 0,033	0,816 ± 0,033
6	0,773 ± 0,037	0,774 ± 0,037	0,790 ± 0,036	0,786 ± 0,036	0,778 ± 0,036	0,781 ± 0,036
7	0,683 ± 0,042	0,739 ± 0,040	0,726 ± 0,040	0,723 ± 0,040	0,741 ± 0,039	0,744 ± 0,039
8	0,685 ± 0,042	0,684 ± 0,043	0,686 ± 0,042	0,720 ± 0,040	0,724 ± 0,040	0,730 ± 0,039
9	0,609 ± 0,045	0,642 ± 0,043	0,688 ± 0,042	0,701 ± 0,041	0,708 ± 0,040	0,732 ± 0,039
10	0,624 ± 0,044	0,680 ± 0,042	0,695 ± 0,041	0,709 ± 0,040	0,737 ± 0,039	0,735 ± 0,039
11	0,691 ± 0,041	0,695 ± 0,041	0,706 ± 0,041	0,734 ± 0,039	0,727 ± 0,040	
12	0,670 ± 0,042	0,683 ± 0,042	0,722 ± 0,040	0,711 ± 0,041		
13	0,649 ± 0,043	0,707 ± 0,040	0,694 ± 0,042			
14	0,700 ± 0,040	0,677 ± 0,043				
15	0,605 ± 0,045					

The area under the ROC-curve (AUC) was highest for bin size 4, trials 3 to 6.

**Figure 2:**
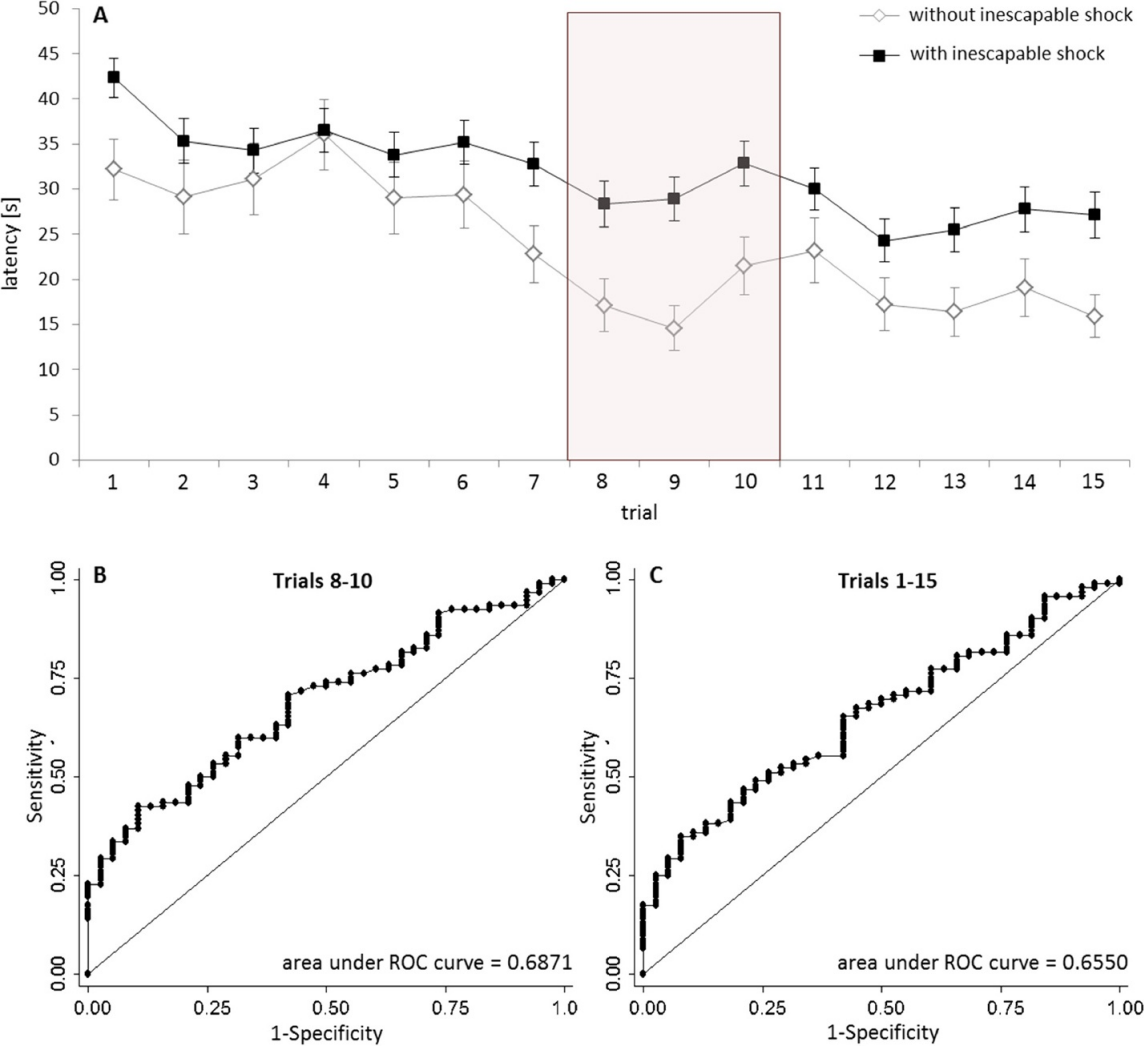
**Reanalysis of data from 130 Sprague Dawley rats tested for learned helplessness with (n = 92) or without prior exposition to inescapable shock (n = 38). A**: Latency until pressing the lever across the 15 trials of the test for learned helplessness is presented separately for the groups (without inescapable shock, with inescapable shock) as means ± SEM. As indicated by the red box, the latency difference between animals with and without inescapable shock was most pronounced in trials 8 to 10. **B** and **C**: ROC-curves: Latencies measurements of the trials 8 to 10 discriminated animals with and without inescapable shock with the same sensitivity and specificity as trials 1 to 15 did.

**Figure 3:**
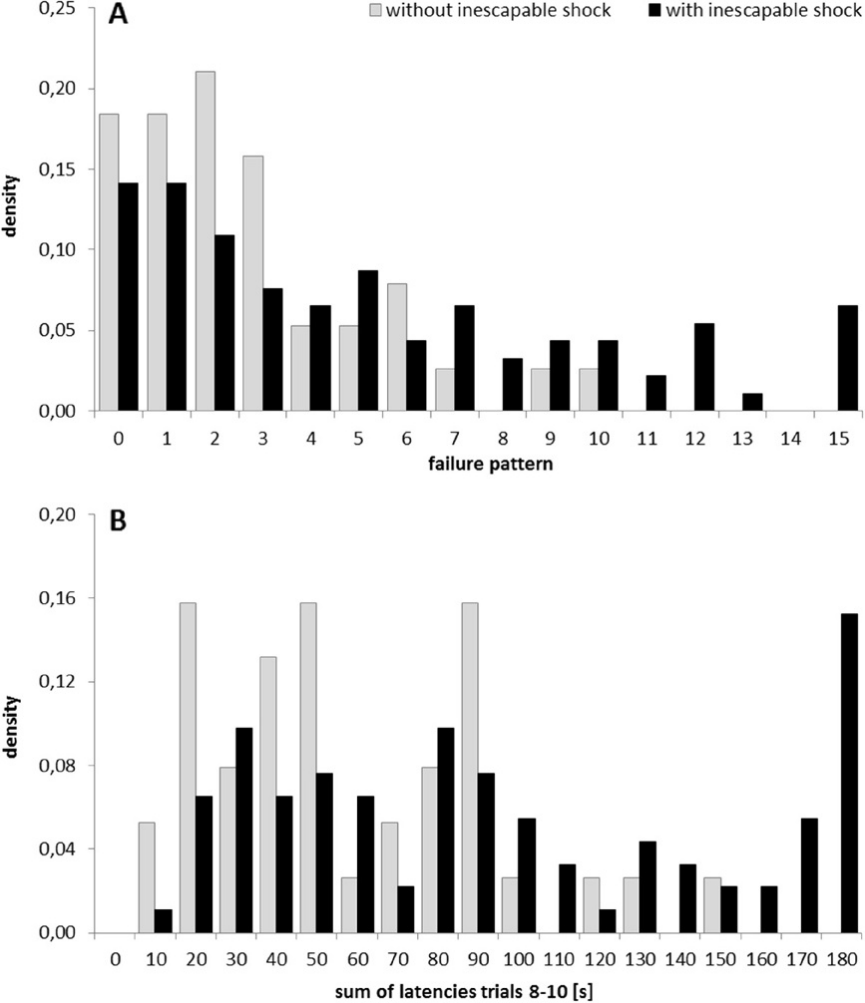
**Distribution densities of failure patterns and latencies of 130 Sprague Dawley rats tested for learned helplessness with (n = 92, black bars) or without prior exposition to inescapable shock (n = 38, grey bars). A**: Our LH paradigm uses inescapable shock of relatively low intensity. Consequently, behavioral variation is high and most of the exposed rats do not become helpless. Up to date the number of failures to press the lever out of 15 trials shock has been accepted as the ‘gold standard’ to discriminate a small fraction of helpless rats after inescapable shock. Rats not exposed to inescapable shock have served as a control and determined the cut-off criterion for helplessness to be ≥ 11. **B**: Distribution of sum of latencies only from trials 8–10 identified the cut-off criterion for helplessness to be ≥ 150 s and yielded a better separation of the helpless fraction among the rats tested after inescapable shock.

In a second step, we reanalysed LH-tests from 130 male Sprague Dawley outbred rats [11]. Out of the 130 rats, 82 were tested 24 h after inescapable shock, while 38 were tested without previous shock exposure. In comparison to cLH and cNLH rats, differences in response times between rats with or without previous shock exposure seemed to be less prominent over the course of LH testing (Figure 2A). However, the descriptive analysis suggested the difference between the two rat groups to be most prominent between trials 8 and 10 (Figure 2A). To investigate variation in statistical power across the trials statistically, we again varied the bin size from one to four and used sliding windows to maximize the area under the ROC-curve. As expected on the basis of the descriptive analysis, the AUC was highest for trials 8 to 10 (bin size 3, Figure 2B). Again, the absolute AUC value was slightly higher for trials 8 to 10 than for all trials (Figure 2C), indicating a statistical power at least as high as in the widely accepted 15-trial-procedure. Because overall distinctness is more subtle in wildtype rats than in cLH/cNLH rats, limiting the analysis to those trials that guarantee high discriminative power, may thus even improve the identification of different subgroups. Indeed, when compared to the failure pattern, the widely used “gold standard” in the field, the sum of latencies across trials 8 to 10 seemed to better distinguish the helpless subgroup from the non-helpless majority (Figure 3). The analysis of latency measures thus provides a highly sensitive method to reliably differentiate between helpless and non-helpless rats and may further contribute to the refinement of animal experimentation in the best of meanings of the 3R-concept [16].

## Conclusions

Taken together, re-analyses of LH tests of both wildtype Sprague Dawley and selectively bred cLH and cNLH rats revealed a trial-dependent statistical power for the classification of helpless and non-helpless animals. Thus, over the course of the 15 trials considerable behavioral changes in latency measurements became evident: While the first one or two trials were characterized by unspecific reactions, statistical power increased in the subsequent trials, reached a maximum between trials 3 and 10, and was less pronounced in the last third of the testing session. The existence of such prominent behavioral changes over the course of the trials clearly argues for the use of a continuous measure in the analysis of LH behavior and favours the integration of a trial-by-trial analysis that benefits the identification of those trials that have the most statistical power, thus improving overall sensitivity and specificity of the approach.

From an animal-ethical point of view, a refinement of the procedure is highly desirable. Since our findings indicate that statistical power is best between trials 3 and 10 and lowest in the last third of trials, the procedure can be shortened from 15 to 10 trials to reduce pain experience and refine the procedure as far as possible.
